# Aptamer-based search for correlates of plasma and serum water T_2_: implications for early metabolic dysregulation and metabolic syndrome

**DOI:** 10.1186/s40364-018-0143-x

**Published:** 2018-09-17

**Authors:** Vipulkumar Patel, Alok K. Dwivedi, Sneha Deodhar, Ina Mishra, David P. Cistola

**Affiliations:** 1 Nanoparticle Diagnostics Laboratory, Institute for Cardiovascular & Metabolic Diseases, University of North Texas Health Science Center, Fort Worth, TX 76107 USA; 2 Center of Emphasis in Diabetes & Metabolism, Paul L. Foster School of Medicine, Texas Tech University Health Sciences Center El Paso, El Paso, TX 79905 USA; 3 Division of Biostatistics & Epidemiology, Paul L. Foster School of Medicine, Texas Tech University Health Sciences Center El Paso, El Paso, TX 79905 USA

**Keywords:** Metabolic syndrome, Insulin resistance, Nuclear magnetic resonance relaxometry, Transverse relaxation time constant, Hepatocyte growth factor, Glucokinase regulatory protein, Receptor tyrosine kinase FLT3, SOMAscan aptamer assay, Random forests, Classification and regression tree analysis

## Abstract

**Background:**

Metabolic syndrome is a cluster of abnormalities that increases the risk for type 2 diabetes and atherosclerosis. Plasma and serum water T_2_ from benchtop nuclear magnetic resonance relaxometry are early, global and practical biomarkers for metabolic syndrome and its underlying abnormalities. In a prior study, water T_2_ was analyzed against ~ 130 strategically selected proteins and metabolites to identify associations with insulin resistance, inflammation and dyslipidemia. In the current study, the analysis was broadened ten-fold using a modified aptamer (SOMAmer) library, enabling an unbiased search for new proteins correlated with water T_2_ and thus, metabolic health.

**Methods:**

Water T_2_ measurements were recorded using fasting plasma and serum from non-diabetic human subjects. In parallel, plasma samples were analyzed using a SOMAscan assay that employed modified DNA aptamers to determine the relative concentrations of 1310 proteins. A multi-step statistical analysis was performed to identify the biomarkers most predictive of water T_2_. The steps included Spearman rank correlation, followed by principal components analysis with variable clustering, random forests for biomarker selection, and regression trees for biomarker ranking.

**Results:**

The multi-step analysis unveiled five new proteins most predictive of water T_2_: hepatocyte growth factor, receptor tyrosine kinase FLT3, bone sialoprotein 2, glucokinase regulatory protein and endothelial cell-specific molecule 1. Three of the five strongest predictors of water T_2_ have been previously implicated in cardiometabolic diseases. Hepatocyte growth factor has been associated with incident type 2 diabetes, and endothelial cell specific molecule 1, with atherosclerosis in subjects with diabetes. Glucokinase regulatory protein plays a critical role in hepatic glucose uptake and metabolism and is a drug target for type 2 diabetes. By contrast, receptor tyrosine kinase FLT3 and bone sialoprotein 2 have not been previously associated with metabolic conditions. In addition to the five most predictive biomarkers, the analysis unveiled other strong correlates of water T_2_ that would not have been identified in a hypothesis-driven biomarker search.

**Conclusions:**

The identification of new proteins associated with water T_2_ demonstrates the value of this approach to biomarker discovery. It provides new insights into the metabolic significance of water T_2_ and the pathophysiology of metabolic syndrome.

**Electronic supplementary material:**

The online version of this article (10.1186/s40364-018-0143-x) contains supplementary material, which is available to authorized users.

## Background

Metabolic syndrome (MetS) is a cluster of clinical findings that includes increased waist circumference, high blood pressure, high blood glucose, high triglycerides and/or low HDL-cholesterol [1, 2]. The criteria for MetS differ depending on the guideline used, but a widely used consensus requires that at least three of the five criteria are met [3]. Metabolic syndrome is associated with a two-fold increased risk for atherosclerosis, a five-fold increased risk for type 2 diabetes [2] and an increased risk for some forms of cancer [4]. The prevalence of MetS is high among the U.S. population: one third of adults and half of those ≥60 years of age [5, 6]. Previously called insulin resistance syndrome [7], the pathophysiological factors that drive MetS include insulin resistance, inflammation and ectopic lipid deposition [1, 2].

In an observational cross-sectional study of 72 non-diabetic human subjects, we discovered that plasma and serum water T_2_ detect MetS-associated abnormalities with high sensitivity and specificity [8]. Measured using benchtop nuclear magnetic resonance relaxometry [9], T_2_ refers to the time constant for the decay or “relaxation” of the transverse component of the NMR signal. Water T_2_ is sensitive to the rotational diffusion of protein-bound and unbound water molecules and serves as a surveillance system for shifts in blood proteins and lipoproteins. One example is the shifts that occur with an acute phase response, which increase the levels of some globulins, while decreasing albumin. As globulins are higher molecular weight than albumin, the net effect is to slow the rotational mobility of bound water and decrease water T_2_ [8, 9].

Fasting hyperinsulinemia (insulin resistance), dyslipidemia and inflammation each have independent and additive contributions to the lowering of water T_2_ [8]. Hence, water T_2_ captures a global view of an individual’s metabolic health status with just one measurement. It shows promise as a screening test for the early detection of poor metabolic health to prevent diabetes and cardiovascular disease [8]. However, the role of water T_2_ in probing metabolic health and elucidating the pathophysiology of MetS has not been fully explored.

The initial search for metabolic correlates of water T_2_ was conducted using 130 strategically-selected blood biomarkers that measure different aspects of metabolic health status [8]. Biomarker selection was based on investigator-driven hypotheses and priorities. While the prior search yielded a wealth of information, it could have been limited by selection bias. Therefore, the search for new correlates of water T_2_ was broadened 10-fold to probe a random library of 1310 plasma proteins using a DNA-based modified aptamer assay developed by SomaLogic, Inc. In this manuscript, we report the results of the SOMAscan analysis of plasma samples from non-diabetic subjects who participated in Phase 2 of the prior study.

Target-specific single-stranded DNA aptamers can be generated in a relatively short time and with substantially less cost than antibodies. Therefore, this technology is gaining recognition as a tool for biomarker discovery [10–18]. A major advantage is that aptamer-based assays are highly multiplexed and can measure hundreds-to-thousands of proteins from biofluids without the need for isolation or pre-treatment [10].

While a broad evaluation of biomarkers is important, it creates challenges related to high-dimension data analysis on a comparatively small number of subjects. Given the large number of proteins measured, the use of statistical correlation alone to identify associations with water T_2_ would increase the probability of false positives. To circumvent this problem, we applied a systematic multi-step method for dimension reduction starting with bivariate correlations, followed by principal components analysis with variable clustering, random forest variable selection, and classification and regression tree analysis or CART. The results identified new predictors of plasma and serum water T_2_ and provided new insights into biomarkers for metabolic health.

## Methods

### Human subject recruitment, blood collection and processing

Human subject research was performed under a protocol approved by the Institutional Review Board of the University of North Texas Health Sciences Center, Fort Worth. A screening interview was completed by each subject prior to obtaining informed consent, and a full medical history was obtained after enrollment. The inclusion criteria were adults ages 18 and up, weighing at least 110 pounds. The exclusion criteria were active acute or chronic illness (history/diagnosis or CRP ≥10), diabetes (history/diagnosis or fasting glucose ≥125 mg/dl or HbA1c ≥6.5%), confirmed or suspected pregnancy, history of bleeding disorders or difficulty giving blood, or not fasting for at least 12 h.

The fasting blood draw was scheduled for 7:00 AM. During the visit, the nurse-phlebotomist recorded routine physical measurements such as height, weight, abdominal waist circumference and blood pressure. In addition, a urine sample was analyzed for microalbuminuria using Chemstrip Micral (Roche Diagnostics, Inc.). The blood samples were centrifuged right after venipuncture using a two-step procedure [8]. For NMR analysis, the freshly drawn and centrifuged samples were analyzed immediately. For SOMAscan assays, the plasma obtained from Phase 2 subjects was biobanked at − 80°C for several months prior to analysis.

### Benchtop NMR relaxometry measurements

The ^1^H NMR data for plasma and serum samples were recorded using a Bruker mq20 Minispec benchtop relaxometer operating at 0.47 T, corresponding to 20 MHz for ^1^H. Samples were pipetted into a 3 mm coaxial insert inside of a 10 mm NMR tube (Norell NI10CCI-B, Norell, Inc., Morganton, North Carolina, USA). The sample height was 1 cm, corresponding to a total volume of ~ 50 μL. A modified Carr-Purcell-Meiboom-Gill (CPMG) pulse sequence was used for T_2_ measurement, as detailed elsewhere [8, 9]. The recycle delay was set to 5 x T_1_ to achieve essentially complete spin relaxation prior to the next round of the pulse sequence. Sixteen scans were signal averaged in each experiment, for a data collection time of 3 min. The data were collected in triplicate. To extract and resolve T_2_ values, the raw CPMG decay curves were analyzed using a discrete inverse Laplace transform algorithm as implemented in XpFit [9, 19]. The number of exponential terms was fixed to three for all samples. Water T_2_ was the dominant term, accounting for > 90% of the total CPMG signal intensity [9].

### SOMAscan proteomics assay

Frozen biobanked plasma samples were shipped overnight on dry ice to SomaLogic, Inc. (Boulder, Colorado, USA) for SOMAscan analysis. The relative concentrations of 1310 plasma proteins were quantified using a proprietary SOMAscan proteomics assay [10]. This assay is based on the selective binding of single-stranded nucleic acid aptamers called SOMAmers (Slow Off-rate Modified Aptamers) to target proteins. The SOMAmer library for target selection was developed using the SELEX method [20, 21].

### Statistical analysis strategy

**Fig. 1:**
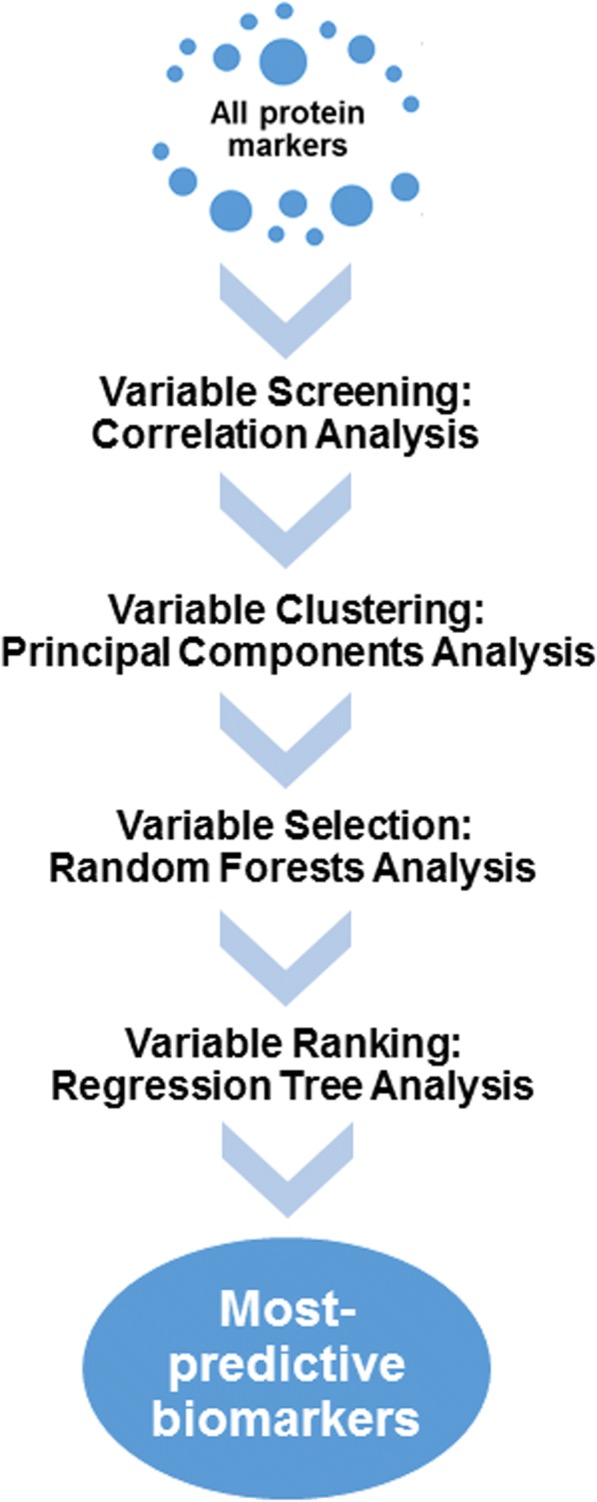
Overall strategy used to identify protein markers in human plasma that are most predictive of plasma or serum water T_2_ values and hence, metabolic health

The search for SOMAscan-detected proteins most predictive of water T_2_ was carried out in four steps: (1) screening the variables using bivariate correlations between protein biomarkers and plasma or serum water T_2_, (2) grouping the correlated proteins into statistically-related clusters and identifying the most representative variable in each cluster, (3) selecting the most predictive variables using an iterative multi-variable random forests analysis, and (4) defining the interactions of the most predictive biomarkers and their final associations with plasma or serum water T_2_ levels. The general scheme is illustrated in Fig. 1, and each of the steps is explained further below.

### Correlation and variable cluster analysis

First, the 1310 SOMAscan-derived biomarkers were analyzed using the Shapiro-Wilk normality test in R 3.1.4 statistical software [22]. Based on this analysis, only 408 (~ 31%) of the variables followed a normal distribution. Therefore, the correlations of plasma or serum T_2_ with SOMAscan-derived biomarkers were screened using non-parametric Spearman rank correlation coefficients (ρ values). The screening criterion was |ρ| ≥ 0.3. In the preselection of variables associated with water T_2_, we focused on the effect size (correlation coefficient), not statistical significance (*p*-value), in order to limit false negatives.

To reduce the dimensionality of the search at the screening stage, we applied principal components analysis with variable clustering as implemented in JMP Pro 12.1.0 (SAS, Inc., Cary, North Carolina, USA). The algorithm identified variable clusters, as well as the most representative variable in each cluster [23]. Variable clustering is not to be confused with conventional cluster analysis, which identifies clustering across subjects, as opposed to clustering across measured variables. It has advantages over factor analysis for dimension reduction and has been recently used in clinical research [24]. In addition, variable clustering reduces the difficulty in interpreting the output of conventional principal component analysis [23]. For each cluster, the variable corresponding to the largest squared correlation with its cluster component was identified as the most representative variable and used for the next step of statistical analysis.

### Random forests and CART analysis

The most representative variables from all clusters were used as independent variables, and water T_2_ as a dependent variable, to construct two random forest models: one for plasma water T_2_ and another for serum water T_2_. Random forests, developed by Leo Breiman and colleagues, is a powerful non-parametric machine learning algorithm to make predictions from the data [25]. In addition, it can be used as a tool to select variables, in this case SOMAscan-derived protein markers, based on their importance in predicting plasma or serum water T_2_. This analysis was performed using the package randomForest in R 3.1.4 [22, 26].

The randomForest algorithm generated regression trees based on a statistical resampling or bootstrap method. It started with a randomly-selected subset of the original data, i.e., a learning set containing approximately one third of the protein variables. Each learning set was used to create a regression tree, where the first branch contained the protein variable that showed the maximum difference in T_2_ between the two branches, with an approximately equal number of subjects in each branch. Similarly, additional branches were created until each variable in the learning set was incorporated into the tree. Through bootstrapping, a total of 1000 regression trees were created from 1000 randomly-selected learning sets. This method ensured the stability of the results by repeating the association analysis a large number of times. For each subject, the T_2_ value predicted from all 1000 trees was averaged and compared to the experimentally determined T_2_ for that subject. Finally, the mean squared error was calculated by comparing the predicted vs. observed T_2_ values across *all* subjects.

To select the most predictive variables, the random forests analysis was repeated after removing all trees containing a given variable. Then the remaining trees were used to predict the T_2_ value for a given subject, and the mean squared error was calculated to quantify predicted vs. observed T_2_ across all subjects. This process was performed recursively by leaving out trees containing one variable at a time and calculating a new mean squared error. The *percent change* in mean squared error before and after leaving out each variable was computed, and the variables were ranked by the percent change. By convention, protein variables with ≥5% change in mean squared error after being removed from the random forests model were selected as the top predictors of water T_2_. Note that the use of the 5% threshold was somewhat arbitrary, and proteins falling just below this threshold also are predictive of water T_2_.

Using the most predictive variables, two final regression trees were constructed using classification and regression tree analysis or CART: one for plasma and one for serum water T_2_. The CART analysis explores the possible interactions across all the selected variables by determining the most appropriate binary classification of each variable. The regression trees were constructed by identifying variables that maximized the T_2_ difference while keeping the number of subjects in each branch approximately equal. The branching was stopped when the number of subjects in each branch was < 25% of the total number of subjects in the study.

### Multiple regression analysis

As a cross check on the most predictive variables identified by random forest, the variables were used to generate multiple linear regression models, with plasma or serum water T_2_ as the outcome variable. The models were constructed using the stepwise tools in JMP Pro v14.0, and acceptable models met the following criteria [8]: (1) all predictor variables were statistically significant at α = 0.05, (2) the models were not overfit, as assessed by k-fold cross validation, and (3) the adjusted R^2^ was maximized.

## Results

### Characteristics of the human subject cohort

The study population consisted of asymptomatic individuals without active acute or chronic disease (Table 1). There were approximately equal numbers of males and females. The mean values for clinical lab tests fell within their reference ranges, although some individuals had values outside the normal range. By American Diabetes Association criteria, 15 of the 41 Phase 2 subjects had prediabetes based on HbA_1c_ and/or fasting glucose levels; none had overt diabetes. Using the harmonized criteria [3], 9 of 41 subjects met the definition of MetS. By water T_2_ criteria, 19 of the 41 subjects had hyperinsulinemia/insulin resistance using the cut points established by Robinson et al. [8]. Five of the 19 (26%) had compensatory hyperinsulinemia (early metabolic dysregulation) and did not meet the criteria for either prediabetes or MetS.

**Table 1:** Characteristics of the human study population (*n* = 41)

Parameter	Mean ± S.D.	Range	Reference Values^a^
Age	36.5 ± 12.5	23–61	n/a
Gender	n/a	19 female, 22 male	n/a
Body-Mass Index (kg/m^2^)	26.5 ± 5.5	19.1–45.1	< 25 normal weight, 25–30 overweight, > 30 obese
Plasma T_2_ (ms)	771.5 ± 58.3	631–887	≥ 745.0^b^
Serum T_2_ (ms)	817.4 ± 5.2	706–908	≥ 811.8^b^
Glucose (mg/dL)	90.6 ± 7.7	71–109	< 100 non-diabetic
			100–125 (pre-diabetic)
HbA_1c_ (%)	5.5 ± 0.3	4.7–6.1	< 5.7 (non-diabetic)
			5.7–6.4 (pre-diabetic)
Insulin C-peptide (ng/mL)	1.8 ± 0.8	0.7–5.1	0.8–3.9 (> 2.85, IR^c^)
Insulin (μU/mL)	8.7 ± 6.6	2.2–40.1	2.0–19.6 (> 12.2, IR^c^)
Total serum protein (g/dL)	7.2 ± 0.4	6.3–8.0	6.1–8.1
Serum albumin (g/dL)	4.5 ± 0.3	3.6–5.1	3.6–5.1
Serum globulins (g/dL)	2.7 ± 0.3	1.9–3.3	1.9–3.7
Triglycerides (mg/dL)	123 ± 63.1	50–321	< 150
Total cholesterol (mg/dL)	185.0 ± 45.0	97–291	< 200
HDL-C (mg/dL)	51.7 ± 12.7	31–78	≥ 40 (male); ≥ 50 (female)
LDL-C (mg/dL)	110.1 ± 35.9	50–191	< 130
WBC count (× 10^3^/μL)	6.6 ± 1.7	3.9–11.2	3.8–10.8
Neutrophil count (× 10^3^/μL)	3.6 ± 1.2	1.8–7.2	1.5–7.8
*hs*-CRP (mg/L)	2.6 ± 2.7	0.1–9.6	< 3.0 (low/average CV risk)
			3.0–10.0 (high CV risk)
			> 10.0 (infection/illness)
Sodium (mmoles/L)	138.2 ± 2.6	131–143	135–146
Potassium (mmoles/L)	4.1 ± 0.3	3.7–4.8	3.5–5.3
Total CO_2_, (mmoles/L)	24.2 ± 2.3	18–28	19–30

^a^Reference values from Quest Diagnostics and Atherotech, except where noted

^b^Cutoff for normoglycemic population established in previous study [8]

^c^Insulin cutoff from McAuley et al. [67]; insulin C-peptide cutoff established by linear regression with insulin

### Bivariate correlations and variable clustering analysis

**Fig. 2:**
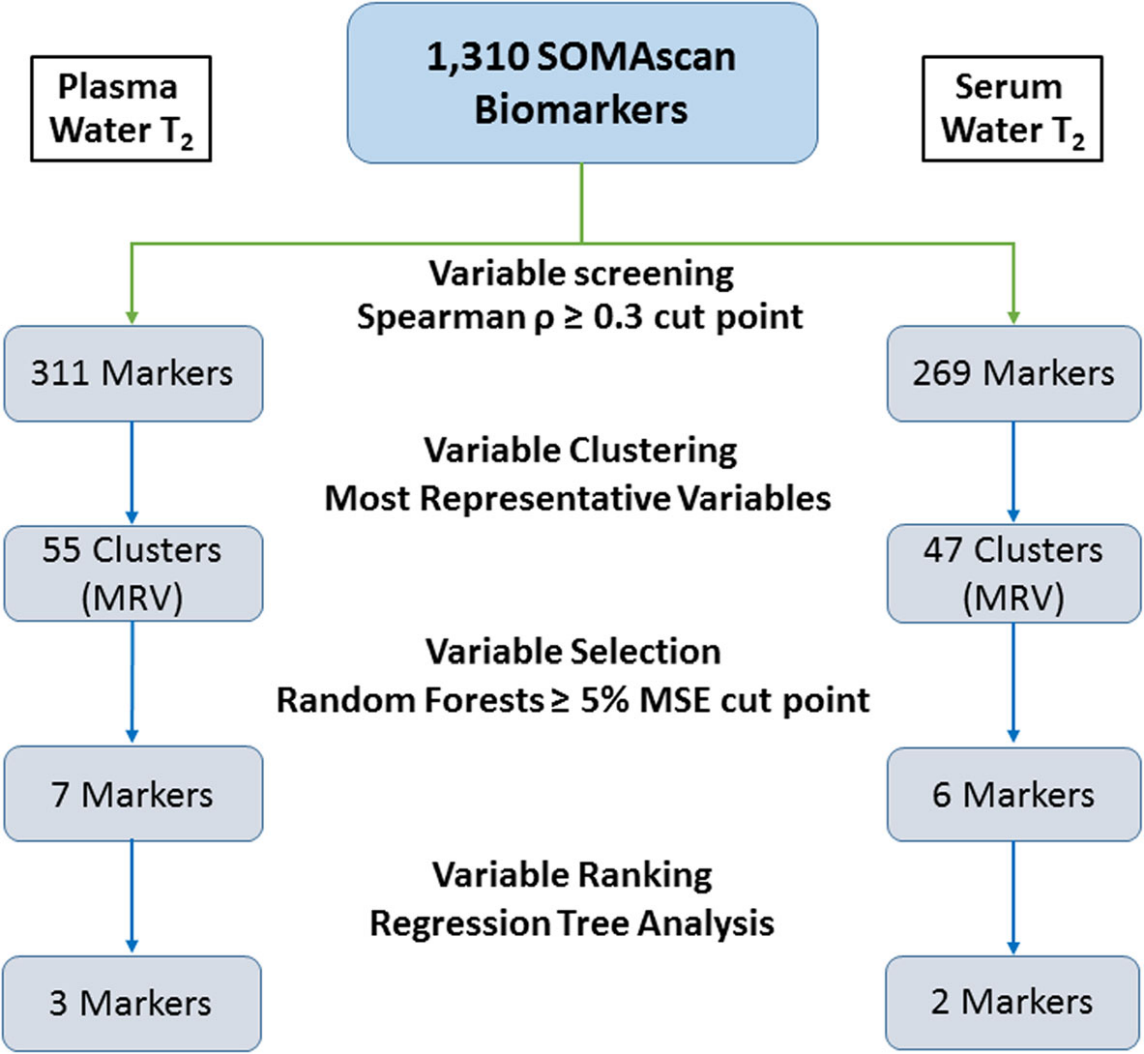
Numbers of SOMAscan-derived protein biomarkers identified at each stage of the data analysis. The left branch shows the analysis results for plasma water T_2_, and the right branch, serum water T_2_. MRV, most representative variable; MSE, mean squared error

Figure 2 provides a schematic overview of the results from each stage of statistical analysis for plasma (left side) and serum water T_2_ (right side). The correlation analysis revealed 311 and 269 protein markers for plasma and serum T_2_, respectively, using a Spearman ρ absolute-value threshold of 0.3. The full lists of 311 and 269 protein markers with correlation coefficients are provided in Additional file 1: Tables S1 and S2, respectively.

The correlated variables were further subjected to dimension reduction using variable clustering. The clustering algorithm revealed 55 and 47 clusters for plasma and serum water T_2_, respectively. Additional file 1: Tables S3 and S4 list all of the clusters, as defined by their most representative variables, for plasma and serum T_2_-correlated biomarkers, respectively.

### Random forests and CART analysis

The most representative variable from each cluster was selected for random forests analysis. This analysis yielded 7 proteins most predictive for plasma water T_2_ (Table 2) and 6 for serum water T_2_ (Table 3). Each protein displayed a percent increase in mean squared error ≥ 5% after trees containing this protein were removed from the random forests model. As shown in Tables 2 and 3, glucokinase regulatory protein and receptor-type tyrosine protein kinase FLT3 were top predictors of *both* plasma and serum water T_2_.

**Table 2:** Most predictive biomarkers and cluster members for plasma water T_2_

Protein Name (Uniprot ID)^a^	% Inc. MSE^a^	Cluster Members
Hepatocyte growth factor (P14210)	9.51	R-spondin-2, Galectin-7
Glucokinase regulatory protein (Q14397)	9.44	Low-density lipoprotein receptor-related protein 1 soluble, T-lymphocyte activation antigen CD86,
Receptor-type tyrosine-protein kinase FLT3 (P36888)	8.34	Complement C4b^b^, Discoidin domain-containing receptor 2, Serine/threonine-protein kinase PAK 6, Heterogeneous nuclear ribonucleoprotein A/B
Ephrin-B2 (P52799)	7.52	Leucine-rich repeat transmembrane protein FLRT3, Amphoterin-induced protein 2, Ephrin-A5,NT-3 growth factor receptor, Kallikrein-8, Interleukin-1 receptor type 1, Iduronate 2-sulfatase, CD109 antigen, Cell adhesion molecule 1, SLIT and NTRK-like protein 5, Ephrin type-A receptor 2, Endoglin, Interleukin-22 receptor subunit alpha-2, OX-2 membrane glycoprotein, Semaphorin-6B, Semaphorin-6A, Interferon alpha/beta receptor 1
Bone sialoprotein 2 (P21815)	5.22	Fibrinogen^b^, Alpha-1-antichymotrypsin, Antithrombin-III, Endothelial cell-specific molecule 1, Serotransferrin
Histone-lysine N-methyl-transferase EHMT2 (Q96KQ7)	5.13	Metalloproteinase inhibitor 1, Metalloproteinase inhibitor 2, Delta-like protein 4
Fibroblast growth factor 2 (P09038)	5.09	Fibroblast growth factor 4

^a^The most predictive biomarkers are defined as those with ≥5% increase in mean squared error (MSE)

^b^In a prior study, complement C4 (C4c) and fibrinogen were strongly associated with plasma water T_2_ [8]

**Table 3:** Most predictive biomarkers and cluster members for serum water T_2_

Protein Name (Uniprot ID)^a^	% Inc. MSE^a^	Cluster Members
Endothelial cell-specific molecule 1 (Q9NQ30)	11.0	Bone sialoprotein 2, Bone morphogenetic protein 10
Glucokinase regulatory protein (Q14397)	8.0	2′-5′-oligoadenylate synthase 1, T-lymphocyte activation antigen CD86
Lactadherin (Q08431)	7.4	Hepatocyte growth factor receptor, Alpha-2-macroglobulin, Adrenomedullin, N terminal pro BNP
Vascular cell adhesion protein 1 (P19320)	6.1	Secreted frizzled-related protein 3, L-selectin
Receptor-type tyrosine-protein kinase FLT3 (P36888)	5.6	Serum amyloid P-component, Discoidin domain-containing receptor 2, Serine/threonine-protein kinase PAK 6, Heterogeneous nuclear ribonucleoprotein A/B
Semaphorin-6A (Q9H2E6)	5.2	Osteopontin, Leucine-rich repeat transmembrane protein FLRT3, Ephrin-B2, Ephrin-A5, NT-3 growth factor receptor, Kallikrein-8, Interleukin-1 receptor type 1, Iduronate 2-sulfatase, CD109 antigen, Cell adhesion molecule 1, Brother of CDO, SLIT and NTRK-like protein 5, Endoglin, Neuropilin-1

^a^The most predictive biomarkers are defined as those with ≥5% increase in mean squared error (MSE)

**Fig. 3:**
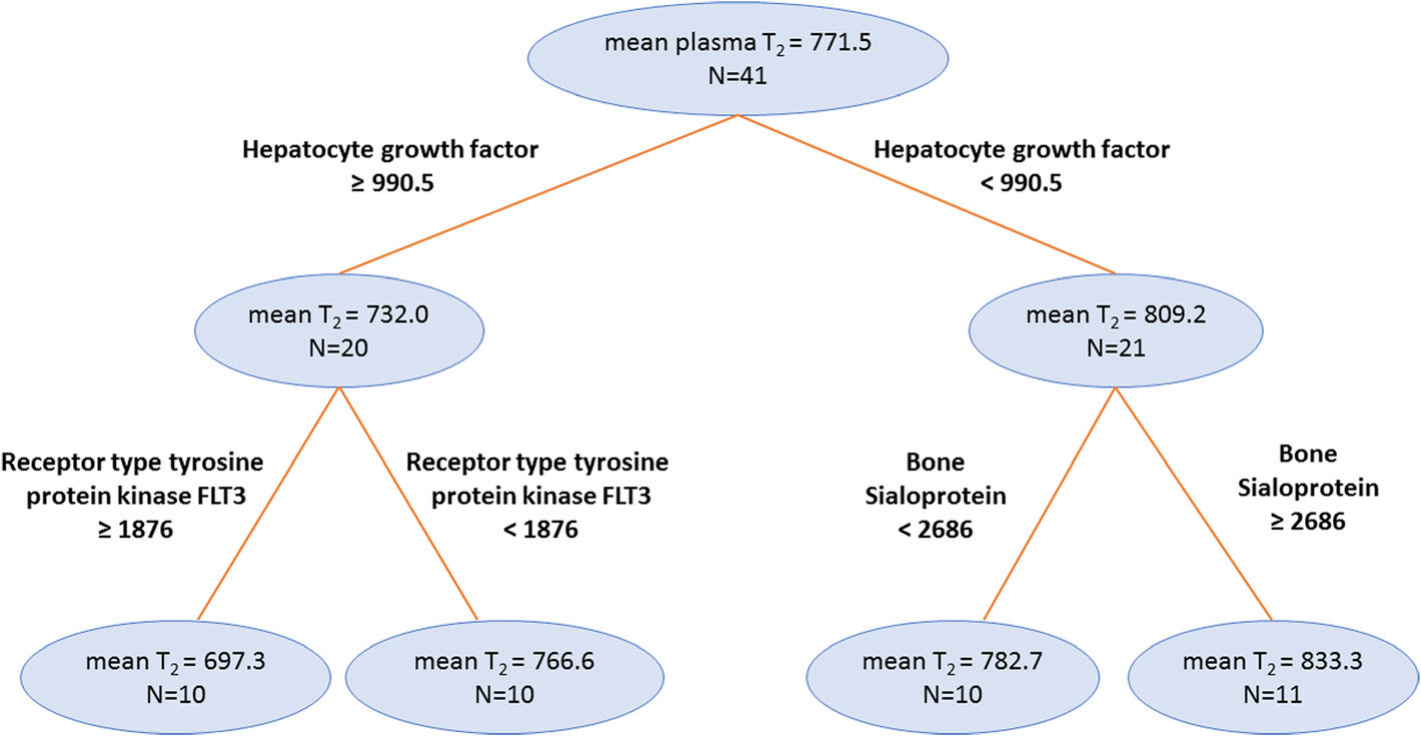
Final regression tree showing the protein biomarkers most predictive for plasma water T_2_. The mean plasma water T_2_ values are in milliseconds, and the SOMAscan protein biomarker cut points are in relative units. The number of subjects (N) in each branch is indicated

**Fig. 4:**
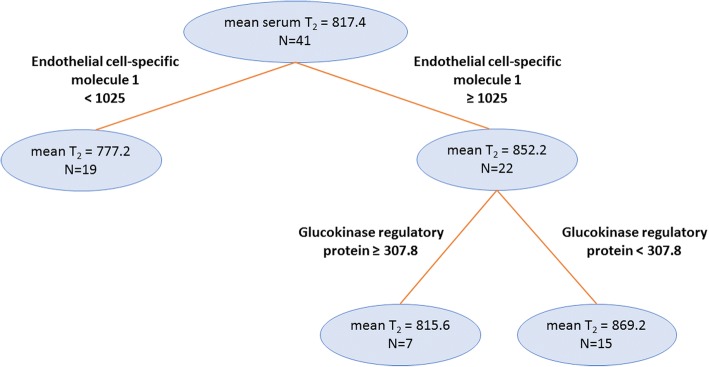
Final regression tree showing the protein biomarkers most predictive for serum water T_2_. The mean serum water T_2_ values are in milliseconds, and the SOMAscan protein biomarker cut points are in relative units. The number of subjects (N) in each branch is indicated

As revealed by CART analysis, the final regression tree for plasma water T_2_ included three biomarkers: hepatocyte growth factor receptor, receptor tyrosine kinase FLT3 (fms-like tyrosine kinase 3), and bone sialoprotein 2 (Fig. 3). The final regression tree for serum water T_2_ included two protein markers: endothelial cell-specific molecule 1 and glucokinase regulatory protein (Fig. 4).

### Multiple regression analysis

As a validation check for the random forest results, we tested the variables listed in Tables 2 and 3 as predictor variables in multiple linear regression models, with plasma or serum water T_2_ as the outcome variable. The best model for plasma water T_2_ incorporated hepatic growth factor, receptor-type tyrosine protein kinase FLT3 and bone sialoprotein, yielding an adjusted R^2^ of 0.52. These three predictor variables accounted for over half of the variation in plasma water T_2_. For serum water T_2_, the best model incorporated endothelial cell specific molecule 1, receptor-type tyrosine protein kinase FLT3 and semaphorin 6A, yielding an adjusted R^2^ of 0.47. Thus, the results from random forests are consistent with those obtained from a different method.

## Discussion

For the first time, a highly multiplexed SOMAscan assay was used in an unbiased search for new correlates of plasma and serum water T_2_. Using this discovery strategy, we identified proteins in the SOMAmer library that were most predictive of water T_2_ and hence, metabolic health [8]. The dimensionality was reduced using a systematic multi-step procedure that incorporated principal components analysis with variable clustering, random forests, and classification and regression trees. The analysis unveiled five proteins most predictive of plasma and serum water T_2_, as well as six other proteins that emerged from the random forests analysis as strong predictors. All are new hits, as none of these proteins were included or considered in the prior hypothesis-driven biomarker search for correlates of water T_2_.

Three proteins were most predictive of plasma water T_2_: hepatocyte growth factor, receptor tyrosine kinase FLT3 (fms-like tyrosine kinase 3) and bone sialoprotein 2. The latter two proteins have not been previously associated with metabolic conditions or diabetes. However, FLT3 is implicated in inflammation, immunity and autoimmune diseases and is overexpressed in leukemia [27, 28]. Also known as CD135, FLT3 is involved in development of immune cells in bone marrow and peripheral lymphoid tissue [29, 30]. In particular, FLT3 regulates the growth of hematopoietic stem cells and the development/homeostasis of dendritic cells in lymphoid tissue [29, 30]. Activation of the receptor by mutation leads to proliferation, resistance to apoptosis and prevention of differentiation, leading to myeloid leukemia.

Hepatic growth factor has been implicated in diabetes-related conditions [31–35]. It is elevated in overt type 2 diabetes [35] as well as diabetes-associated coronary artery disease and cerebral infarction [31, 33]. Most relevant to the current study are recent results from the multi-ethnic study of atherosclerosis (MESA), a longitudinal human cohort study. The MESA results revealed that *elevated levels of HGF predict incident type 2 diabetes* [36]. The current observation of a strong inverse association between plasma water T_2_ and HGF is consistent with this finding, as low water T_2_ detects early metabolic conditions thought to lead to type 2 diabetes, namely insulin resistance, subclinical inflammation and dyslipidemia [8]. In addition, water T_2_ is strongly and inversely correlated with complement C3, C4, fibrinogen, and haptoglobin, markers predictive of incident type 2 diabetes [8, 37].

The hepatic growth factor *receptor*, also known as MET, is part of a tyrosine kinase signaling complex that functions in cell growth and survival, angiogenesis and tissue regeneration [38–40]. It is expressed in cells of mesenchymal origin, including epithelial and endothelial cells, neurons, hepatocytes, adipocytes, myocytes and pancreatic cells. The receptor is cell-membrane associated (c-MET), but a soluble ectodomain (s-MET) is shed and circulates in plasma [41]. The receptor is upregulated in cancer, and both c-MET and s-MET have been investigated as biomarkers of malignancy, metastasis and tumor progression [40, 42–44].

In this study, s-MET (soluble HGF receptor) displayed positive Spearman correlations with plasma and serum water T_2_ (+ 0.45 and + 0.44, respectively; *p* < 0.01; Additional file 1: Tables S1 and S2). Those correlations were opposite in sign to those for the receptor ligand HGF. Like HGF, MET was among the variables predictive of water T_2_, but at 4.2%, was just below the 5% mean-squared error threshold employed in Tables 2 and 3. Thus, high HGF and low soluble HGF receptor are associated with low water T_2_ and poor metabolic health.

In the pancreas, HGF/MET signaling is necessary for beta-cell regeneration [45]. A pancreas-specific knockout of the MET gene in mice accelerates the onset of diabetes [46]. Also, hepatocyte growth factor signaling is thought to be a mediator of beta cell proliferation in obesity [47]. Moreover, hypoxia-inducible factor (HIF1), which is associated with obesity and sleep apnea, is a transcriptional regulator of MET [48–50]. Thus, the expression of MET appears to be increased under conditions of metabolic dysregulation that place high secretory demand on beta cells, such as obesity, insulin resistance and tissue hypoxia. A decreased ability to upregulate MET under these conditions may hasten the demise of beta cells and accelerate the onset of type 2 diabetes.

The current observation of an association between plasma water T_2_ and HGF/MET reinforces the notion that low plasma water T_2_ is a biomarker of metabolic dysregulation and poor metabolic health, even in individuals without prediabetes or metabolic syndrome [8]. Given the association of plasma water T_2_ with other proteins that predict future type 2 diabetes and atherosclerosis, namely fibrinogen, complement C3 and C4, haptoglobin, α1-acid glycoprotein (orosomucoid) and apolipoprotein B, the association of water T_2_ with HGF provides further evidence that plasma water T_2_ is a biomarker of the metabolic dysregulation that precedes type 2 diabetes and cardiovascular disease [8].

Bone sialoprotein 2, named according to its high sialic acid content, is expressed during the development of bone and cementum [51]. The function of this protein is unknown but believed to serve as a nucleation site for hydroxyapatite crystals [52]. Expression of this protein is regulated by hormones, growth factors and cytokines [53]. As shown in Table 2, fibrinogen is a member of this protein cluster (Table 2) and likely mediates the statistical association between plasma water T_2_ and bone sialoprotein 2. Fibrinogen is the fourth most abundant protein in plasma. Changes in its level directly affects plasma water T_2_ [8]. Endothelial cell specific molecule 1 is in that cluster as well.

The CART regression tree analysis for *serum* water T_2_ yielded two biomarkers: endothelial cell specific molecule 1 and glucokinase regulatory protein (GKRP). Endothelial cell specific molecule 1 (ESM-1 or endocan) is involved in angiogenesis and plays a role in lung-endothelial cell-leukocyte interactions [54, 55]. It has recently been implicated in subclinical atherosclerosis in type II diabetes patients [56]. In addition, ESM-1 is involved or implicated in prostate cancer [57], endothelial injury in respiratory distress syndrome [58], oral cancer [59], erectile dysfunction [60], and pulmonary infection [61]. Note that the ESM-1 cluster for serum water T_2_ includes bone sialoprotein 2, but not fibrinogen (Table 3). Serum water T_2_ is unaffected by fibrinogen levels, as this protein is absent in serum.

Glucokinase regulatory protein is a well-known inhibitor of glucokinase and a key regulator of liver glucose uptake and metabolism [62–65]. Normally, GKRP is an intracellular protein localized within hepatocytes. As shown here, increased GKRP levels in plasma and serum were associated with a lowering of T_2_ values and a worsening of metabolic health, specifically insulin resistance and glucose intolerance. This observation implies that GKRP is leaking from hepatocytes into the circulation, perhaps reflective of early liver damage. None of the subjects in this study have a history of liver disease, but that does not rule out the possibility of subclinical hepatic steatosis or steatohepatitis. This interpretation is supported by the positive correlation between GKRP and ALT observed in these subjects (Spearman ρ = 0.47, *p* = 0.0024). Alanine aminotransferase (ALT) is an established marker of liver damage. Plasma and serum T_2_ are correlated with both GKRP (this study) and ALT [8].

### Study limitations

For two reasons, this study utilized a relatively small number of subjects. First, biobanked samples were available only from Phase 2 of the initial biomarker discovery study for plasma and serum water T_2_ [8]. Second, the SomaScan analysis was expensive, placing practical constraints on its application. At first glance, a small sample size might lead to concerns about statistical power. However, power depends not only on sample size, but also effect size. In this study, the effect size was remarkably large for the most predictive variables identified by random forest. A power calculation for *N* = 41 revealed that a power of 0.8 would be achieved for absolute values of correlation coefficients >|0.425| at α = 0.05. By comparison, the Spearman coefficient for hepatocyte growth factor and plasma water T_2_ was − 0.52, and the Huber M-value correlation was − 0.67. Likewise, the Spearman and Huber correlation coefficients for endothelial cell specific protein 1 and serum water T_2_ were 0.58 and 0.70, respectively. Thus, the current analysis was sufficiently powered because of the large effect sizes, which more than compensated for the relatively small N.

The small sample size placed a practical lower limit on the initial biomarker screening step, possibly generating false negatives by failing to detect some biomarkers that are more weakly, but significantly associated with water T_2_. Therefore, future studies with larger N may unveil additional biomarkers that are less predictive, but still significant contributors to water T_2_. Also, a future study with a different group of subjects will be important for validating the most predictive variables discovered in the current study.

The NMR analysis was performed using freshly-drawn plasma and serum. However, the SOMAscan analysis was performed, by necessity, using one-time frozen-thawed biobanked plasma. Changes in some plasma proteins may have occurred during the freeze-thaw process and could have impacted the analysis. Such changes, if occurred, were likely to be minor, as biobanked plasma and serum are generally stable after one freeze-thaw cycle [66].

## Conclusion

The SOMAscan results and multi-stage regression analyses yielded new correlates and predictors of plasma and serum water T_2_ that were not previously identified in a hypothesis-driven biomarker search. These new predictors broadened our understanding of the biomarker network and the information content of plasma and serum water T_2_. In addition, the discovery of biomarkers correlated with water T_2_ provided new insights into the pathophysiology of metabolic syndrome and the early metabolic dysregulation that precedes type 2 diabetes and cardiovascular disease.

## Additional file

Additional file 1: **Table S1.** Spearman correlation of plasma water T_2_ with SOMAscan biomarkers. **Table S2.** Spearman correlation of serum water T_2_ with SOMAscan biomarkers. **Table S3.** Clusters of plasma T_2_-correlated SOMAscan biomarkers. **Table S4.** Clusters of serum T_2_-correlated SOMAscan biomarkers. (DOCX 86 kb)

## Abbreviations

ADA: American Diabetes Association
AHA: American Heart Association
CART: Classification and regression tree
CPMG: Carr-Purcell-Meiboom-Gill pulse sequence used to measure T_2_
ESM-1: Endothelial cell-specific molecule 1
FLT3: fms-like tyrosine kinase 3
GKRP: Glucokinase regulatory protein
HGF: Hepatocyte growth factor
MESA: Multi-ethnic Study of Atherosclerosis
MetS: Metabolic syndrome
MSE: Mean squared error
NMR: Nuclear magnetic resonance
SELEX: Systematic evolution of ligands by exponential enrichment
ss-DNA: Single stranded DNA
T_2_: Transverse relaxation time
