# Supplementary material for: Aptamer-based search for correlates of plasma and serum water T2: implications for early metabolic dysregulation and metabolic syndrome
Source: Biomark Res. 2018 Sep 17;6:28. doi: 10.1186/s40364-018-0143-x (PMC6142358; doi:10.1186/s40364-018-0143-x)
Supplement: Supplementary file 1 — Table S1. Spearman correlation of plasma water T2 with SOMAscan biomarkers. Table S2. Spearman correlation of serum water T2 with SOMAscan biomarkers. Table S3. Clusters of plasma T2-correlated SOMAscan biomarkers. Table S4. Clusters of serum T2-correlated SOMAscan biomarkers. (DOCX 86 kb) [file 40364_2018_143_MOESM1_ESM.docx]

**Table S1. Spearman correlation of plasma water T_2_ with SOMAscan biomarkers**

| Protein Name | Uniprot ID | Spearman’s ρ |
| --- | --- | --- |
| Endothelial cell-specific molecule 1 | Q9NQ30 | 0.76**** |
| Ephrin-B2 | P52799 | 0.65**** |
| Histone H3.1 | P68431 | 0.62**** |
| Neuropilin-1 | O14786 | 0.59**** |
| Bone morphogenetic protein 1 | P13497 | -0.59**** |
| Insulin-like growth factor-binding protein 2 | P18065 | 0.59**** |
| Semaphorin-6A | Q9H2E6 | 0.58**** |
| Glucokinase regulatory protein | Q14397 | -0.57**** |
| Antithrombin-III | P01008 | 0.57*** |
| NT-3 growth factor receptor | Q16288 | 0.56*** |
| Endoglin | P17813 | 0.56*** |
| Osteopontin | P10451 | 0.55*** |
| Layilin | Q6UX15 | 0.55*** |
| Bone sialoprotein 2 | P21815 | 0.55*** |
| Wnt inhibitory factor 1 | Q9Y5W5 | 0.54*** |
| Somatotropin | P01241 | -0.54*** |
| Gamma-enolase | P09104 | 0.54*** |
| CD59 glycoprotein | P13987 | 0.54*** |
| Phosphatidylethanolamine-binding protein 1 | P30086 | -0.53*** |
| Ubiquitin-40S ribosomal protein S27a | P62979 | -0.53*** |
| 72 kDa type IV collagenase | P08253 | 0.52*** |
| Alpha-1-antichymotrypsin | P01011 | 0.52*** |
| Histone-lysine N-methyltransferase EHMT2 | Q96KQ7 | 0.52*** |
| Hepatocyte growth factor | P14210 | -0.52*** |
| Cell adhesion molecule 1 | Q9BY67 | 0.52*** |
| A disintegrin and metalloproteinase with thrombospondin motifs 15 | Q8TE58 | -0.51*** |
| Interferon alpha/beta receptor 1 | P17181 | 0.51*** |
| Fibroblast growth factor 19 | O95750 | 0.51*** |
| Delta-like protein 4 | Q9NR61 | 0.51*** |
| RGM domain family member B | Q6NW40 | 0.51*** |
| Ephrin-A5 | P52803 | 0.51*** |
| Ficolin-3 | O75636 | -0.51*** |
| Amphoterin-induced protein 2 | Q86SJ2 | 0.50** |
| Bone morphogenetic protein 10 | O95393 | 0.50*** |
| Trefoil factor 1 | P04155 | 0.50** |
| Leucine-rich repeat transmembrane protein FLRT3 | Q9NZU0 | 0.49** |
| Tyrosine-protein kinase JAK2 | O60674 | 0.49** |
| C-C motif chemokine 16 | O15467 | -0.48** |
| Dickkopf-like protein 1 | Q9UK85 | -0.48** |
| Protein Name | **Uniprot ID** | **Spearman’s ρ** |
| Tumor necrosis factor receptor superfamily member 11B | O00300 | 0.48** |
| Tissue-type plasminogen activator | P00750 | -0.48** |
| Interleukin-17F | Q96PD4 | -0.47** |
| Carbonic anhydrase 2 | P00918 | -0.47** |
| Low-density lipoprotein receptor-related protein 1B | Q9NZR2 | -0.47** |
| C-C motif chemokine 2 | P13500 | -0.47** |
| C5a anaphylatoxin | P01031 | -0.47** |
| Complement component C1q receptor | Q9NPY3 | 0.47** |
| OX-2 membrane glycoprotein | P41217 | 0.47** |
| Biglycan | P21810 | 0.47** |
| Natriuretic peptides B | P16860 | -0.47** |
| Tissue factor pathway inhibitor | P10646 | -0.47** |
| Cytoskeleton-associated protein 2 | Q8WWK9 | 0.47** |
| Tumor necrosis factor-inducible gene 6 protein | P98066 | 0.47** |
| Roundabout homolog 3 | Q96MS0 | -0.47** |
| Mitogen-activated protein kinase 9 | P45984 | 0.46** |
| Protein 4.1 | P11171 | -0.46** |
| Hepatocyte growth factor receptor | P08581 | 0.46** |
| Interleukin-17A | Q16552 | -0.46** |
| CD109 antigen | Q6YHK3 | 0.46** |
| Phospholipase A2 | P04054 | 0.46** |
| Vesicular integral-membrane protein VIP36 | Q12907 | -0.46** |
| L-selectin | P14151 | 0.46** |
| Prolactin | P01236 | 0.45** |
| T-lymphocyte activation antigen CD86 | P42081 | 0.45** |
| Agouti-related protein | O00253 | 0.45** |
| Tumor necrosis factor receptor superfamily member 8 | P28908 | 0.45** |
| Proteasome subunit alpha type-2 | P25787 | 0.45** |
| Iduronate 2-sulfatase | P22304 | 0.45** |
| Ck-beta-8-1 | P55773 | 0.45** |
| Interleukin-10 | P22301 | -0.45** |
| C-X-C motif chemokine 5 | P42830 | -0.45** |
| Hsp90 co-chaperone Cdc37 | Q16543 | -0.45** |
| Insulin-like growth factor-binding protein 1 | P08833 | 0.45** |
| Complement C1s subcomponent | P09871 | -0.45** |
| Fatty acid-binding protein, heart | P05413 | -0.45** |
| Interleukin-1 receptor type 1 | P14778 | 0.45** |
| Carbonic anhydrase 4 | P22748 | 0.44** |
| Leptin | P41159 | -0.44** |
| Activin receptor type-1B | P36896 | -0.44** |
| Interleukin-1 receptor like 2 | Q9HB29 | 0.44** |
| Neurogenic locus notch homolog protein 1 | P46531 | 0.44** |
| Protein Name | **Uniprot ID** | **Spearman’s ρ** |
| Inactive tyrosine-protein kinase transmembrane receptor ROR1 | Q01973 | 0.44** |
| Ubiquitin-conjugating enzyme E2 G2 | P60604 | -0.44** |
| Lactadherin | Q08431 | 0.44** |
| Choline/ethanolamine kinase | Q9Y259 | -0.43** |
| Discoidin domain-containing receptor 2 | Q16832 | 0.43** |
| Interleukin-13 | P35225 | -0.43** |
| Delta-like protein 1 | O00548 | 0.43** |
| Proteasome subunit alpha type-1 | P25786 | -0.43** |
| Erythropoietin receptor | P19235 | -0.43** |
| Vascular cell adhesion protein 1 | P19320 | 0.42** |
| Microtubule-associated protein tau | P10636 | -0.42** |
| Kallikrein-11 | Q9UBX7 | 0.42** |
| Laminin | P25391, P07942, P11047 | -0.42** |
| C3a anaphylatoxin | P01024 | -0.42** |
| Complement C5b C6 complex | P01031,P13671 | -0.42** |
| Serotransferrin | P02787 | 0.42** |
| Complement factor H | P08603 | -0.42** |
| Adenylate kinase isoenzyme 1 | P00568 | -0.41** |
| Tumor necrosis factor ligand superfamily member 15 | O95150 | 0.41** |
| Quinone oxidoreductase-like protein 1 | O95825 | -0.41** |
| 26S proteasome non-ATPase regulatory subunit 7 | P51665 | 0.41** |
| Cell adhesion molecule 3 | Q8N126 | 0.41** |
| Alpha-2-macroglobulin | P01023 | 0.41** |
| Eukaryotic initiation factor 4A-III | P38919 | -0.41** |
| Interleukin-22 receptor subunit alpha-2 | Q969J5 | 0.41** |
| Retinoic acid receptor responder protein 2 | Q99969 | -0.41** |
| P-selectin | P16109 | -0.41** |
| Serum paraoxonase/arylesterase 1 | P27169 | -0.41** |
| Fatty acid-binding protein, epidermal | Q01469 | -0.41** |
| Leucine-rich repeat transmembrane protein FLRT2 | O43155 | 0.41** |
| Brother of CDO | Q9BWV1 | 0.41** |
| Opioid-binding protein/cell adhesion molecule | Q14982 | -0.40** |
| SLAM family member 5 | Q9UIB8 | 0.40** |
| Platelet-derived growth factor receptor alpha | P16234 | 0.40** |
| Metalloproteinase inhibitor 2 | P16035 | 0.40** |
| Heparan-sulfate 6-O-sulfotransferase 1 | O60243 | 0.40* |
| Trefoil factor 2 | Q03403 | 0.40* |
| Follistatin | P19883 | -0.40* |
| Semaphorin-6B | Q9H3T3 | 0.40* |
| Protein Name | **Uniprot ID** | **Spearman’s ρ** |
| Heterogeneous nuclear ribonucleoproteins A2/B1 | P22626 | -0.40* |
| Protein amnionless | Q9BXJ7 | -0.40* |
| Complement C5 | P01031 | -0.40* |
| Brain-derived neurotrophic factor | P23560 | -0.40* |
| Sex hormone-binding globulin | P04278 | 0.40* |
| Natural cytotoxicity triggering receptor 1 | O76036 | 0.40* |
| R-spondin-2 | Q6UXX9 | -0.40* |
| CD70 antigen | P32970 | -0.40* |
| Cytochrome c | P99999 | 0.40* |
| Kallikrein-8 | O60259 | 0.39* |
| Insulin-like growth factor 1 receptor | P08069 | 0.39* |
| Parathyroid hormone-related protein | P12272 | 0.39* |
| Insulin-like growth factor-binding protein 7 | Q16270 | 0.39* |
| Interleukin-17D | Q8TAD2 | -0.39* |
| Serum albumin | P02768 | 0.39* |
| Cytotoxic and regulatory T-cell molecule | O95727 | -0.39* |
| Azurocidin | P20160 | -0.39* |
| C-C motif chemokine 5 | P13501 | -0.39* |
| Galectin-7 | P47929 | -0.39* |
| Plasminogen | P00747 | -0.39* |
| Metalloproteinase inhibitor 3 | P35625 | -0.39* |
| Megakaryocyte-associated tyrosine-protein kinase | P42679 | -0.39* |
| Interferon gamma receptor 2 | P38484 | -0.39* |
| Histone acetyltransferase type B catalytic subunit | O14929 | -0.39* |
| A disintegrin and metalloproteinase with thrombospondin motifs 4 | O75173 | -0.39* |
| Serum amyloid P-component | P02743 | -0.39* |
| Serine/threonine-protein kinase 16 | O75716 | 0.39* |
| Stromelysin-1 | P08254 | -0.39* |
| T-lymphocyte surface antigen Ly-9 | Q9HBG7 | 0.39* |
| Interleukin-1 Receptor accessory protein | Q9NPH3 | 0.39* |
| Glial cell line-derived neurotrophic factor | P39905 | -0.38* |
| Complement C3b | P01024 | -0.38* |
| Low-density lipoprotein receptor-related protein 8 | Q14114 | 0.38* |
| ICOS ligand | O75144 | 0.38* |
| Interleukin-11 | P20809 | -0.38* |
| Granzyme H | P20718 | -0.38* |
| Fibroblast growth factor 2 | P09038 | 0.38* |
| Carboxypeptidase E | P16870 | 0.38* |
| Insulin-like growth factor-binding protein 4 | P22692 | -0.38* |
| Rab GDP dissociation inhibitor beta | P50395 | -0.38* |
| Heterogeneous nuclear ribonucleoprotein A/B | Q99729 | -0.38* |
| Protein Name | **Uniprot ID** | **Spearman’s ρ** |
| Junctional adhesion molecule B | P57087 | 0.38* |
| Ectonucleoside triphosphate diphosphohydrolase 1 | P49961 | 0.38* |
| Pituitary adenylate cyclase-activating polypeptide | P18509 | -0.37* |
| Ephrin type-A receptor 2 | P29317 | 0.37* |
| cGMP-dependent 3',5'-cyclic phosphodiesterase | O00408 | -0.37* |
| Killer cell lectin-like receptor subfamily F member 1 | Q9NZS2 | -0.37* |
| Ubiquitin carboxyl-terminal hydrolase 25 | Q9UHP3 | -0.37* |
| Fibroblast growth factor 18 | O76093 | -0.37* |
| Creatine kinase B-type | P12277 | -0.37* |
| Growth arrest-specific protein 1 | P54826 | 0.37* |
| Vascular endothelial growth factor A isoform 121 | P15692 | -0.37* |
| Angiopoietin-1 | Q15389 | -0.37* |
| Interleukin-6 | P05231 | -0.36* |
| Calcineurin subunit B type 1 | P63098 | -0.36* |
| N-terminal pro-BNP | P16860 | 0.36* |
| Mitochondrial import inner membrane translocase subunit TIM14 | Q96DA6 | -0.36* |
| Coactosin-like protein | Q14019 | 0.36* |
| Prostaglandin G/H synthase 2 | P35354 | -0.36* |
| Thrombospondin-1 | P07996 | -0.36* |
| Platelet-derived growth factor subunit B | P01127 | -0.36* |
| Insulin-like growth factor binding protein 6 | P24592 | 0.36* |
| Complement decay-accelerating factor | P08174 | 0.36* |
| Secreted-frizzled related protein 3 | Q92765 | 0.36* |
| Plasminogen activator inhibitor 1 | P05121 | -0.36* |
| Macrophage migration inhibitory factor | P14174 | -0.36* |
| Interleukin-18 receptor accessory protein | O95256 | -0.36* |
| Protein S100-A4 | P26447 | 0.36* |
| Neutrophil activating peptide-2, Platelet basic protein | P02775 | -0.36* |
| Dynactin subunit 2 | Q13561 | -0.36* |
| Apolipoprotein E | P02649 | -0.36* |
| Latent-transforming growth factor beta binding protein 4 | Q8N2S1 | 0.36* |
| Insulin-degrading enzyme | P14735 | 0.36* |
| C-C motif chemokine 24 | O00175 | -0.36* |
| Lysosomal protective protein | P10619 | -0.36* |
| Connective tissue-activating peptide III | P02775 | -0.35* |
| Estrogen receptor | P03372 | -0.35* |
| Endoplasmic reticulum resident protein 29 | P30040 | -0.35* |
| C-C motif chemokine 20 | P78556 | -0.35* |
| Complement factor I | P05156 | -0.35* |
| Lipopolysaccharide-binding protein | P18428 | 0.35* |
| Insulin-like growth factor I | P05019 | 0.35* |
| Protein Name | **Uniprot ID** | **Spearman’s ρ** |
| Alpha-synuclein | P37840 | -0.35* |
| Insulin | P01308 | -0.35* |
| Transforming growth factor beta receptor type 3 | Q03167 | 0.35* |
| Ubiquitin-conjugating enzyme E2 N | P61088 | -0.35* |
| Bactericidal permeability-increasing protein | P17213 | -0.35* |
| Interferon gamma | P01579 | 0.35* |
| Contactin-1 | Q12860 | 0.35* |
| Non-receptor tyrosine-protein kinase TYK2 | P29597 | -0.35* |
| Nicotinamide phosphoribosyltransferase | P43490 | -0.35* |
| Gro-beta/gamma | P19876 P19875 | -0.35* |
| Cytokine receptor-like factor 1:Cardiotrophin-like cytokine factor 1 Complex | O75462 Q9UBD9 | -0.35* |
| Osteocalcin | P02818 | 0.35* |
| Complement C3b, inactivated | P01024 | -0.35* |
| Carbonic anhydrase 3 | P07451 | -0.35* |
| C-C motif chemokine 28 | Q9NRJ3 | -0.35* |
| Neuronal growth regulator 1 | Q7Z3B1 | 0.34* |
| Follistatin-related protein 1 | Q12841 | 0.34* |
| Semaphorin-3A | Q14563 | -0.34* |
| Asialoglycoprotein receptor 1 | P07306 | 0.34* |
| Platelet-derived growth factor C | Q9NRA1 | -0.34* |
| Cadherin-5 | P33151 | 0.34* |
| Interleukin-23 | P29460, Q9NPF7 | 0.34* |
| SPARC | P09486 | -0.34* |
| Fibroblast growth factor receptor 1 | P11362 | 0.34* |
| SPARC-related modular calcium-binding protein 1 | Q9H4F8 | 0.34* |
| Group IIE secretory phospholipase A2 | Q9NZK7 | -0.34* |
| Endothelial cell-selective adhesion molecule | Q96AP7 | 0.34* |
| Mannan-binding lectin serine protease 1 | P48740 | 0.34* |
| Kallikrein-5 | Q9Y337 | 0.34* |
| SLIT and NTRK-like protein 5 | O94991 | 0.34* |
| Advanced glycosylation end product-specific receptor soluble | Q15109 | 0.34* |
| Vitamin K-dependent protein S | P07225 | -0.34* |
| Troponin I, fast skeletal muscle | P48788 | 0.34* |
| Complement C3 | P01024 | -0.34* |
| Baculoviral IAP repeat-containing protein 3 | Q13489 | -0.34* |
| Low-density lipoprotein receptor | P01130 | -0.33* |
| Kallikrein-7 | P49862 | 0.33* |
| Neuroblastoma suppressor of tumorigenicity 1 | P41271 | 0.33* |
| Netrin receptor UNC5C | O95185 | 0.33* |
| Protein Name | **Uniprot ID** | **Spearman’s ρ** |
| Gelsolin | P06396 | 0.33* |
| Desmoglein-1 | Q02413 | 0.33* |
| Urokinase-type plasminogen activator | P00749 | 0.33* |
| C-C motif chemokine 23 | P55773 | 0.33* |
| Tyrosine-protein kinase ABL1 | P00519 | -0.33* |
| C-reactive protein | P02741 | -0.33* |
| Angiopoietin-related protein 3 | Q9Y5C1 | -0.33* |
| Complement C4b | P0C0L4 P0C0L5 | -0.33* |
| Alpha-2-antiplasmin | P08697 | 0.33* |
| Lysosome membrane protein 2 | Q14108 | -0.33* |
| Placenta growth factor | P49763 | -0.33* |
| Interleukin-4 | P05112 | -0.33* |
| Protein Wnt-7a | O00755 | -0.33* |
| Amyloid-beta A4 protein | P05067 | -0.33* |
| Platelet factor 4 | P02776 | -0.33* |
| Lysozyme C | P61626 | 0.33* |
| CD48 antigen | P09326 | 0.33* |
| Carbonic anhydrase 1 | P00915 | -0.33* |
| DNA repair protein RAD51 homolog 1 | Q06609 | 0.32* |
| Sialic acid-binding Ig-like lectin 14 | Q08ET2 | 0.32* |
| Low-density lipoprotein receptor-related protein 1, soluble | Q07954 | 0.32* |
| Proteasome activator complex subunit 3 | P61289 | -0.32* |
| Brain-specific serine protease 4 | Q9GZN4 | 0.32* |
| Death-associated protein kinase 2 | Q9UIK4 | 0.32* |
| Cathepsin L2 | O60911 | 0.32* |
| Ribosomal protein S6 kinase alpha-5 | O75582 | -0.32* |
| Contactin-4 | Q8IWV2 | 0.32* |
| Stromal cell-derived factor 1 | P48061 | 0.32* |
| Fibroblast growth factor 4 | P08620 | -0.32* |
| Cadherin-6 | P55285 | -0.32* |
| Acid sphingomyelinase-like phosphodiesterase 3a | Q92484 | -0.32* |
| Cytoplasmic tyrosine-protein kinase BMX | P51813 | -0.32* |
| Neurexin-3-beta | Q9HDB5 | 0.32* |
| Platelet-derived growth factor subunit A | P04085 | -0.32* |
| Ephrin type-A receptor 5 | P54756 | 0.32* |
| Lymphotoxin alpha2:beta1 | P01374, Q06643 | 0.32* |
| Myoglobin | P02144 | 0.32* |
| Tumor necrosis factor receptor superfamily member 21 | O75509 | 0.32* |
| Lymphocyte activation gene 3 protein | P18627 | 0.32* |
| Desmocollin-2 | Q02487 | 0.32* |
| Protein Name | **Uniprot ID** | **Spearman’s ρ** |
| Peroxiredoxin-1 | Q06830 | -0.31* |
| Leukotriene A-4 hydrolase | P09960 | 0.31* |
| A disintegrin and metalloproteinase with thrombospondin motifs 5 | Q9UNA0 | -0.31* |
| Fatty acid-binding protein, liver | P07148 | 0.31* |
| Diablo homolog, mitochondrial | Q9NR28 | 0.31* |
| Thymic stromal lymphopoietin | Q969D9 | -0.31* |
| N-acylethanolamine-hydrolyzing acid amidase | Q02083 | 0.31* |
| NudC domain-containing protein 3 | Q8IVD9 | 0.31* |
| Serine/threonine-protein kinase PAK 6 | Q9NQU5 | -0.31* |
| 40S ribosomal protein S7 | P62081 | -0.31* |
| Vitronectin | P04004 | 0.31* |
| Receptor-type tyrosine-protein kinase FLT3 | P36888 | -0.31* |
| Metalloproteinase inhibitor 1 | P01033 | 0.31† |
| Appetite-regulating hormone | Q9UBU3 | 0.31† |
| Protein jagged-2 | Q9Y219 | -0.31† |
| UL16-binding protein 1, NKG2D ligand 1 | Q9BZM6 | -0.31† |
| Homeobox protein NANOG | Q9H9S0 | -0.31† |
| Leucine carboxyl methyltransferase 1 | Q9UIC8 | 0.31† |
| Basal Cell Adhesion Molecule | P50895 | 0.31† |
| PILR alpha-associated neural protein | Q8IYJ0 | 0.30† |
| Ectonucleoside triphosphate diphosphohydrolase 5 | O75356 | 0.30† |
| Toll-like receptor 2 | O60603 | 0.30† |
| Prolactin receptor | P16471 | 0.30† |
| Tyrosine-protein kinase ZAP-70 | P43403 | 0.30† |
| WNT1-inducible-signaling pathway protein 1 | O95388 | -0.30† |
| Aggrecan core protein | P16112 | 0.30† |
| Protein S100-A12 | P80511 | -0.30† |
| Fibrinogen | P02671/75/79 | 0.30† |
| Non-histone chromosomal protein HMG-14 | P05114 | -0.30† |
| Casein kinase II subunit alpha | P68400 | -0.30† |

*p<0.05 **p<0.01 ***p<0.001 ****p<0.0001 †p≥0.05

**Table S2. Spearman correlation of serum water T_2_ with SOMAscan biomarkers**

| Protein Name | Uniprot ID | | | Spearman’s ρ |
| --- | --- | --- | --- | --- |
| Ephrin-A5 | | | P52803 | 0.62**** |
| 72 kDa type IV collagenase | | | P08253 | 0.58**** |
| Endothelial cell-specific molecule 1 | | | Q9NQ30 | 0.58**** |
| Somatotropin | | | P01241 | -0.58**** |
| Microtubule-associated protein tau | | | P10636 | -0.57**** |
| Bone morphogenetic protein 1 | | | P13497 | -0.57**** |
| Ephrin-B2 | | | P52799 | 0.57*** |
| T-lymphocyte activation antigen CD86 | | | P42081 | 0.56*** |
| Glucokinase regulatory protein | | | Q14397 | -0.56*** |
| Netrin receptor UNC5C | | | O95185 | 0.56*** |
| Semaphorin-3E | | | O15041 | 0.55*** |
| Wnt inhibitory factor 1 | | | Q9Y5W5 | 0.54*** |
| Neutrophil gelatinase-associated lipocalin | | | P80188 | 0.54*** |
| Neuropilin-1 | | | O14786 | 0.54*** |
| Roundabout homolog 3 | | | Q96MS0 | -0.54*** |
| Alpha-1-antichymotrypsin | | | P01011 | 0.53*** |
| Layilin | | | Q6UX15 | 0.53*** |
| NT-3 growth factor receptor | | | Q16288 | 0.52*** |
| Ectonucleoside triphosphate diphosphohydrolase 1 | | | P49961 | 0.52*** |
| Vascular cell adhesion protein 1 | | | P19320 | 0.52*** |
| Ck-beta-8-1 | | | P55773 | 0.51*** |
| Semaphorin-6A | | | Q9H2E6 | 0.51*** |
| Endoglin | | | P17813 | 0.51*** |
| Gamma-enolase | | | P09104 | 0.51*** |
| Parathyroid hormone-related protein | | | P12272 | 0.50*** |
| Brother of CDO | | | Q9BWV1 | 0.50*** |
| Serotransferrin | | | P02787 | 0.50*** |
| Interferon alpha/beta receptor 1 | | | P17181 | 0.50*** |
| Insulin-like growth factor-binding protein 2 | | | P18065 | 0.50*** |
| Platelet-derived growth factor C | | | Q9NRA1 | -0.50*** |
| Amphoterin-induced protein 2 | | | Q86SJ2 | 0.49** |
| Interleukin-13 | | | P35225 | -0.48** |
| Neurogenic locus notch homolog protein 1 | | | P46531 | 0.48** |
| Transforming growth factor beta receptor type 3 | | | Q03167 | 0.48** |
| N terminal pro BNP | | | P16860_1 | 0.48** |
| Interleukin-17A | | | Q16552 | -0.47** |
| Neurexin-3-beta | | | Q9HDB5 | 0.47** |
| CD70 antigen | | | P32970 | -0.47** |
| Protein Name | | **Uniprot ID** | | **Spearman’s ρ** |
| Protein kinase C zeta type | | | Q05513 | -0.47** |
| Lactadherin | | | Q08431 | 0.46** |
| Prostaglandin G/H synthase 2 | | | P35354 | -0.46** |
| 40S ribosomal protein S7 | | | P62081 | -0.46** |
| Sex hormone-binding globulin | | | P04278 | 0.46** |
| Bone sialoprotein 2 | | | P21815 | 0.45** |
| L-selectin | | | P14151 | 0.45** |
| Antithrombin-III | | | P01008 | 0.45** |
| Tumor necrosis factor-inducible gene 6 protein | | | P98066 | 0.45** |
| Bone morphogenetic protein 10 | | | O95393 | 0.45** |
| Insulin | | | P01308 | -0.44** |
| Killer cell immunoglobulin-like receptor 3DL2 | | | P43630 | -0.44** |
| Iduronate 2-sulfatase | | | P22304 | 0.44** |
| Leucine-rich repeat transmembrane protein FLRT_2_ | | | O43155 | 0.44** |
| Tyrosine-protein kinase transmembrane receptor ROR1 | | | Q01973 | 0.44** |
| Spectrin alpha chain, non-erythrocytic 1 | | | Q13813 | -0.44** |
| Hepatocyte growth factor receptor | | | P08581 | 0.44** |
| Ubiquitin-conjugating enzyme E2 G2 | | | P60604 | -0.44** |
| Interleukin-11 | | | P20809 | -0.44** |
| Biglycan | | | P21810 | 0.44** |
| Cell adhesion molecule 3 | | | Q8N126 | 0.43** |
| Kallikrein-5 | | | Q9Y337 | 0.43** |
| Tumor necrosis factor receptor superfamily membr 11B | | | O00300 | 0.43** |
| Low-density lipoprotein receptor-related protein 1B | | | Q9NZR2 | -0.43** |
| C-C motif chemokine 23 | | | P55773 | 0.43** |
| Formimidoyltransferase-cyclodeaminase | | | O95954 | -0.43** |
| Gro-beta/gamma | | | P19876/75 | -0.43** |
| Fibroblast growth factor 19 | | | O95750 | 0.43** |
| Muellerian-inhibiting factor | | | P03971 | 0.43** |
| Glial cell line-derived neurotrophic factor | | | P39905 | -0.43** |
| Killer cell lectin-like receptor subfamily F member 1 | | | Q9NZS2 | -0.43** |
| PILR alpha-associated neural protein | | | Q8IYJ0 | 0.43** |
| WAP, Kazal, immunoglobulin, Kunitz and NTR domain-containing protein 2 | | | Q8TEU8 | 0.43** |
| Tumor necrosis factor receptor superfamily member 21 | | | O75509 | 0.42** |
| Serum paraoxonase/arylesterase 1 | | | P27169 | -0.42** |
| Megakaryocyte-associated tyrosine-protein kinase | | | P42679 | -0.42** |
| Carbonic anhydrase 4 | | | P22748 | 0.42** |
| Nuclear receptor subfamily 1 group D member 1 | | | P20393 | -0.42** |
| Discoidin domain-containing receptor 2 | | | Q16832 | 0.42** |
| Protein Name | | | **Uniprot ID** | **Spearman’s ρ** |
| C-C motif chemokine 2 | | | P13500 | -0.42** |
| CD109 antigen | | | Q6YHK3 | 0.42** |
| Cell adhesion molecule 1 | | | Q9BY67 | 0.42** |
| Low-density lipoprotein receptor | | | P01130 | -0.42** |
| Ephrin type-A receptor 2 | | | P29317 | 0.42** |
| Tumor necrosis factor ligand superfamily member 15 | | | O95150 | 0.42** |
| Junctional adhesion molecule B | | | P57087 | 0.41** |
| Carboxypeptidase E | | | P16870 | 0.41** |
| Placenta growth factor | | | P49763 | -0.41** |
| Metalloproteinase inhibitor 2 | | | P16035 | 0.41** |
| Tumor necrosis factor receptor superfamily member 8 | | | P28908 | 0.41** |
| Insulin-like growth factor 1 receptor | | | P08069 | 0.41** |
| Ubiquitin carboxyl-terminal hydrolase 25 | | | Q9UHP3 | -0.41** |
| Interleukin-1 receptor type 1 | | | P14778 | 0.40** |
| Carbonic anhydrase 2 | | | P00918 | -0.40** |
| Lipopolysaccharide-binding protein | | | P18428 | 0.40** |
| Baculoviral IAP repeat-containing protein 3 | | | Q13489 | -0.40** |
| Insulin-degrading enzyme | | | P14735 | 0.40** |
| Persephin | | | O60542 | -0.40** |
| Macrophage migration inhibitory factor | | | P14174 | -0.40** |
| OX-2 membrane glycoprotein | | | P41217 | 0.40** |
| ICOS ligand | | | O75144 | 0.40** |
| Non-receptor tyrosine-protein kinase TYK2 | | | P29597 | -0.40** |
| Angiopoietin-related protein 3 | | | Q9Y5C1 | -0.40** |
| Heterogeneous nuclear ribonucleoproteins A2/B1 | | | P22626 | -0.40** |
| Histone H3.1 | | | P68431 | 0.40** |
| Fibroblast growth factor 4 | | | P08620 | -0.40** |
| Histone-lysine N-methyltransferase EHMT_2_ | | | Q96KQ7 | 0.40** |
| Phosphatidylethanolamine-binding protein 1 | | | P30086 | -0.40** |
| Protein FAM3D | | | Q96BQ1 | 0.40** |
| Desmocollin-2 | | | Q02487 | 0.40** |
| Latent-transforming growth factor β binding protein 4 | | | Q8N2S1 | 0.40** |
| Quinone oxidoreductase-like protein 1 | | | O95825 | -0.40** |
| Ubiquitin-40S ribosomal protein S27a | | | P62979 | -0.39* |
| Urokinase plasminogen activator surface receptor | | | Q03405 | 0.39* |
| Osteopontin | | | P10451 | 0.39* |
| Serum amyloid P-component | | | P02743 | -0.39* |
| Interleukin-1 receptor-like 2 | | | Q9HB29 | 0.39* |
| C-X-C motif chemokine 5 | | | P42830 | -0.39* |
| Protein Name | | | **Uniprot ID** | **Spearman’s ρ** |
| Dickkopf-related protein 3 | | | Q9UBP4 | 0.39* |
| RGM domain family member B | | | Q6NW40 | 0.39* |
| Delta-like protein 4 | | | Q9NR61 | 0.39* |
| Chordin-like protein 1 | | | Q9BU40 | 0.39* |
| SLIT and NTRK-like protein 5 | | | O94991 | 0.39* |
| Tissue-type plasminogen activator | | | P00750 | -0.39* |
| Legumain | | | Q99538 | -0.39* |
| Limbic system-associated membrane protein | | | Q13449 | 0.39* |
| Ephrin type-A receptor 1 | | | P21709 | 0.38* |
| Nidogen-1 | | | P14543 | -0.38* |
| Ephrin-A4 | | | P52798 | 0.38* |
| Metalloproteinase inhibitor 3 | | | P35625 | -0.38* |
| Pulmonary surfactant-associated protein D | | | P35247 | -0.38* |
| Fibronectin | | | P02751 | -0.38* |
| Gastrin-releasing peptide | | | P07492 | -0.38* |
| Granzyme H | | | P20718 | -0.38* |
| Interleukin-6 | | | P05231 | -0.38* |
| Plasminogen | | | P00747 | -0.38* |
| Adrenomedullin | | | P35318 | 0.38* |
| Bone morphogenetic protein 6 | | | P22004 | 0.38* |
| C5a anaphylatoxin | | | P01031 | -0.38* |
| Natural cytotoxicity triggering receptor 1 | | | O76036 | 0.37* |
| Complement component C1q receptor | | | Q9NPY3 | 0.37* |
| Tyrosine-protein kinase JAK2 | | | O60674 | 0.37* |
| Protein jagged-2 | | | Q9Y219 | -0.37* |
| Growth/differentiation factor 9 | | | O60383 | -0.37* |
| Asialoglycoprotein receptor 1 | | | P07306 | 0.37* |
| Interleukin-22 receptor subunit alpha-2 | | | Q969J5 | 0.37* |
| Heme oxygenase 2 | | | P30519 | -0.37* |
| Creatine kinase M-type/Creatine kinase B-type heterodimer | | | P12277 P06732 | -0.37* |
| Alpha-2-antiplasmin | | | P08697 | 0.37* |
| Oncostatin-M | | | P13725 | 0.36* |
| Interferon gamma | | | P01579 | 0.36* |
| Growth arrest-specific protein 1 | | | P54826 | 0.36* |
| Contactin-1 | | | Q12860 | 0.36* |
| Appetite-regulating hormone | | | Q9UBU3 | 0.36* |
| Interferon alpha-7 | | | P01567 | -0.36* |
| Lysosome membrane protein 2 | | | Q14108 | -0.36* |
| Nidogen-2 | | | Q14112 | -0.36* |
| Protein Name | | | **Uniprot ID** | **Spearman’s ρ** |
| Ciliary neurotrophic factor receptor subunit alpha | | | P26992 | 0.36* |
| Fibroblast growth factor receptor 3 | | | P22607 | -0.36* |
| Protein-tyrosine kinase 6 | | | Q13882 | -0.36* |
| Netrin receptor UNC5D | | | Q6UXZ4 | 0.36* |
| SPARC | | | P09486 | -0.36* |
| Corticotropin | | | P01189 | -0.36* |
| Semaphorin-6B | | | Q9H3T3 | 0.36* |
| Eukaryotic initiation factor 4A-III | | | P38919 | -0.36* |
| Insulin-like growth factor-binding protein 1 | | | P08833 | 0.36* |
| Epidermal growth factor | | | P01133 | -0.36* |
| Heparan-sulfate 6-O-sulfotransferase 1 | | | O60243 | 0.36* |
| 2'-5'-oligoadenylate synthase 1 | | | P00973 | -0.36* |
| Receptor-type tyrosine-protein kinase FLT3 | | | P36888 | -0.36* |
| Acid sphingomyelinase-like phosphodiesterase 3a | | | Q92484 | -0.36* |
| Vitamin K dependent protein S | | | P07225 | -0.36* |
| Protein S100-A4 | | | P26447 | 0.36* |
| Endothelial cell-selective adhesion molecule | | | Q96AP7 | 0.36* |
| Activin receptor type 1B | | | P36896 | -0.35* |
| Apolipoprotein E | | | P02649 | -0.35* |
| Proteasome activator complex subunit 3 | | | P61289 | -0.35* |
| Leucine-rich repeat transmembrane protein FLRT3 | | | Q9NZU0 | 0.35* |
| Cryptic protein | | | P0CG37 | 0.35* |
| Calcium/calmodulin-dependent protein kinase kinase 1 | | | Q8N5S9 | -0.35* |
| Fractalkine | | | P78423 | 0.35* |
| Serine/threonine-protein kinase PAK 6 | | | Q9NQU5 | -0.35* |
| HERV-H LTR-associating protein 2 | | | Q9UM44 | 0.35* |
| Trefoil factor 1 | | | P04155 | 0.35* |
| Resistin | | | Q9HD89 | 0.35* |
| Estrogen receptor | | | P03372 | -0.35* |
| C-C motif chemokine 26 | | | Q9Y258 | -0.35* |
| Programmed cell death 1 ligand 2 | | | Q9BQ51 | 0.34* |
| Desmoglein-1 | | | Q02413 | 0.34* |
| Creatine kinase B-type | | | P12277 | -0.34* |
| Follistatin-related protein 1 | | | Q12841 | 0.34* |
| Ephrin type-B receptor 6 | | | O15197 | 0.34* |
| Phosphoglucomutase-1 | | | P36871 | -0.34* |
| Fibrinogen | | | P02671/75/79 | 0.34* |
| Interleukin-17F | | | Q96PD4 | -0.34* |
| Opioid-binding protein/cell adhesion molecule | | | Q14982 | -0.34* |
| Disintegrin/metalloproteinase w/ thrombospondin motifs 13 | | | Q76LX8 | 0.34* |
| Protein Name | | | **Uniprot ID** | **Spearman’s ρ** |
| Platelet-derived growth factor receptor alpha | | | P16234 | 0.34* |
| Phospholipase A2, membrane associated | | | P14555 | -0.34* |
| 14-3-3 protein epsilon | | | P62258 | 0.34* |
| 40S ribosomal protein S3 | | | P23396 | -0.34* |
| Tissue factor pathway inhibitor | | | P10646 | -0.34* |
| Probetacellulin | | | P35070 | -0.34* |
| Heterogeneous nuclear ribonucleoprotein K | | | P61978 | -0.33* |
| Proteasome subunit alpha type-2 | | | P25787 | 0.33* |
| Leukotriene A-4 hydrolase | | | P09960 | 0.33* |
| R-spondin-2 | | | Q6UXX9 | -0.33* |
| Dickkopf-like protein 1 | | | Q9UK85 | -0.33* |
| Osteocalcin | | | P02818 | 0.33* |
| Calcineurin subunit B type 1 | | | P63098 | -0.33* |
| Homeobox protein NANOG | | | Q9H9S0 | -0.33* |
| UL16-binding protein 1, NKG2D ligand 1 | | | Q9BZM6 | -0.33* |
| Angiotensinogen | | | P01019 | 0.33* |
| Delta-like protein 1 | | | O00548 | 0.33* |
| Ephrin type-B receptor 2 | | | P29323 | 0.33* |
| Interferon gamma receptor 2 | | | P38484 | -0.33* |
| Secreted frizzled-related protein 3 | | | Q92765 | 0.33* |
| SLAM family member 5 | | | Q9UIB8 | 0.33* |
| Ficolin-3 | | | O75636 | -0.33* |
| Natriuretic peptides B | | | P16860 | -0.33* |
| Neuronal growth regulator 1 | | | Q7Z3B1 | 0.33* |
| Basal Cell Adhesion Molecule | | | P50895 | 0.33* |
| Endoplasmic reticulum resident protein 29 | | | P30040 | -0.33* |
| Kallikrein-8 | | | O60259 | 0.33* |
| cGMP-dependent 3',5'-cyclic phosphodiesterase | | | O00408 | -0.33* |
| Leptin | | | P41159 | -0.33* |
| Enteropeptidase | | | P98073 | -0.32* |
| Protein amnionless | | | Q9BXJ7 | -0.32* |
| Lysozyme C | | | P61626 | 0.32* |
| Trefoil factor 2 | | | Q03403 | 0.32* |
| C-C motif chemokine 25 | | | O15444 | -0.32* |
| Interleukin-17 receptor B | | | Q9NRM6 | 0.32* |
| NudC domain-containing protein 3 | | | Q8IVD9 | 0.32* |
| Glutathione S-transferase P | | | P09211 | 0.32* |
| Tumor necrosis factor receptor superfamily member 1B | | | P20333 | 0.32* |
| Tumor necrosis factor ligand superfamily member 18 | | | Q9UNG2 | 0.32* |
| CD166 antigen | | | Q13740 | 0.32* |
| Protein Name | | | **Uniprot ID** | **Spearman’s ρ** |
| N-acylethanolamine-hydrolyzing acid amidase | | | Q02083 | 0.32* |
| Trypsin-1 | | | P07477 | 0.32* |
| Heterogeneous nuclear ribonucleoprotein A/B | | | Q99729 | -0.32* |
| Vitronectin | | | P04004 | 0.31* |
| Sialic acid-binding Ig-like lectin 14 | | | Q08ET_2_ | 0.31* |
| Complement C1s subcomponent | | | P09871 | -0.31* |
| C-C motif chemokine 22 | | | O00626 | -0.31* |
| ATP synthase subunit O, mitochondrial | | | P48047 | -0.31* |
| Bactericidal permeability-increasing protein | | | P17213 | -0.31* |
| Angiopoietin-1 receptor | | | Q02763 | 0.31* |
| Vascular endothelial growth factor A isoform 121 | | | P15692 | -0.31* |
| Alpha-2-macroglobulin | | | P01023 | 0.31* |
| Ephrin-A2 | | | O43921 | 0.31* |
| Ephrin type-A receptor 10 | | | Q5JZY3 | -0.31* |
| Group IIE secretory phospholipase A2 | | | Q9NZK7 | -0.31* |
| Tyrosine-protein kinase receptor TYRO3 | | | Q06418 | 0.31* |
| T-lymphocyte surface antigen Ly-9 | | | Q9HBG7 | 0.31* |
| Low affinity immunoglobulin γ Fc region receptor II-b | | | P31994 | -0.31* |
| Matrilin-2 | | | O00339 | 0.31* |
| Peroxisomal targeting signal 1 receptor | | | P50542 | -0.31* |
| Neurogenic locus notch homolog protein 3 | | | Q9UM47 | 0.31* |
| Glia-derived nexin | | | P07093 | -0.31* |
| Diablo homolog, mitochondrial | | | Q9NR28 | 0.31* |
| Cytokine receptor-like factor 2 | | | Q9HC73 | -0.30† |
| Mannan-binding lectin serine protease 1 | | | P48740 | 0.30† |
| Pappalysin-1 | | | Q13219 | 0.30† |
| Endostatin | | | P39060 | 0.30† |
| Fms-related tyrosine kinase 3 ligand | | | P49771 | -0.30† |
| Cytotoxic and regulatory T-cell molecule | | | O95727 | -0.30† |
| Ubiquitin-40S ribosomal protein S27a | | | P62979 | -0.30† |
| Aspartate aminotransferase, cytoplasmic | | | P17174 | 0.30† |
| C-C motif chemokine 5 | | | P13501 | -0.30† |
| Aminoacylase-1 | | | Q03154 | -0.30† |
| Coactosin-like protein | | | Q14019 | 0.30† |

*p<0.05 **p<0.01 ***p<0.001 ****p<0.0001 †p≥0.05

**Table S3. Clusters of plasma T_2_-correlated SOMAscan biomarkers**

| Cluster | Members | Most Representative Variable | Uniprot ID | Cluster Variation Explained^a^ | Total Variation Explained^b^ |
| --- | --- | --- | --- | --- | --- |
| 2 | 19 | Ephrin-B2 | P52799 | 0.668 | 0.041 |
| 4 | 15 | Platelet-derived growth factor subunit B | P01127 | 0.843 | 0.041 |
| 6 | 12 | Tumor necrosis factor receptor superfamily member 21 | O75509 | 0.587 | 0.023 |
| 5 | 8 | Lymphocyte activation gene 3 protein | P18627 | 0.768 | 0.02 |
| 3 | 8 | Toll-like receptor 2 | O60603 | 0.726 | 0.019 |
| 8 | 9 | Opioid-binding protein/cell adhesion molecule | Q14982 | 0.638 | 0.018 |
| 7 | 7 | CD70 antigen | P32970 | 0.802 | 0.018 |
| 31 | 9 | Ribosomal protein S6 kinase alpha-5 | O75582 | 0.621 | 0.018 |
| 25 | 9 | Gelsolin | P06396 | 0.588 | 0.017 |
| 11 | 8 | C3a anaphylatoxin | P01024 4 | 0.654 | 0.017 |
| 15 | 8 | NudC domain-containing protein 3 | Q8IVD9 | 0.614 | 0.016 |
| 14 | 7 | Tumor necrosis factor receptor superfamily member 11B | O00300 | 0.663 | 0.015 |
| 10 | 8 | Quinone oxidoreductase-like protein 1 | O95825 | 0.58 | 0.015 |
| 9 | 8 | Sex hormone-binding globulin | P04278 | 0.557 | 0.014 |
| 12 | 6 | Adenylate kinase isoenzyme 1 | P00568 | 0.736 | 0.014 |
| 1 | 7 | Hepatocyte growth factor receptor | P08581 | 0.62 | 0.014 |
| 13 | 6 | Angiopoietin-related protein 3 | Q9Y5C1 | 0.719 | 0.014 |
| 45 | 7 | Ck-beta-8-1 | P55773 2 | 0.599 | 0.013 |
| 16 | 6 | Complement factor H | P08603 | 0.656 | 0.013 |
| 46 | 6 | Ubiquitin carboxyl-terminal hydrolase 25 | Q9UHP3 | 0.649 | 0.013 |
| 30 | 5 | Tumor necrosis factor ligand superfamily member 15 | O95150 | 0.777 | 0.012 |
| 20 | 6 | Fatty acid-binding protein, epidermal | Q01469 | 0.625 | 0.012 |
| 29 | 5 | Complement component C1q receptor | Q9NPY3 | 0.7 | 0.011 |
| 17 | 6 | Gamma-enolase | P09104 | 0.581 | 0.011 |
| 19 | 5 | Ubiquitin-conjugating enzyme E2 G2 | P60604 | 0.691 | 0.011 |
| 39 | 5 | Desmoglein-1 | Q02413 | 0.678 | 0.011 |
| 22 | 6 | Kallikrein-7 | P49862 | 0.561 | 0.011 |
| 26 | 6 | Bone sialoprotein 2 | P21815 | 0.561 | 0.011 |
| 43 | 5 | Low-density lipoprotein receptor-related protein 8 | Q14114 | 0.65 | 0.01 |
| 23 | 5 | Protein S100-A12 | P80511 | 0.645 | 0.01 |
| 32 | 6 | Proteasome activator complex subunit 3 | P61289 | 0.537 | 0.01 |
| 41 | 5 | Receptor-type tyrosine-protein kinase FLT3 | P36888 | 0.629 | 0.01 |
| 44 | 5 | Insulin-like growth factor 1 receptor | P08069 | 0.608 | 0.01 |
| 27 | 4 | Protein amnionless | Q9BXJ7 | 0.75 | 0.01 |
| 21 | 5 | Carboxypeptidase E | P16870 | 0.573 | 0.009 |
| 33 | 4 | Histone-lysine N-methyltransferase EHMT_2_ | Q96KQ7 | 0.703 | 0.009 |
| 50 | 5 | Semaphorin-3A | Q14563 | 0.532 | 0.009 |
| 24 | 4 | Latent-transforming growth factor beta binding protein 4 | Q8N2S1 | 0.652 | 0.008 |
| 34 | 3 | Glucokinase regulatory protein | Q14397 | 0.832 | 0.008 |
| 52 | 4 | Insulin-like growth factor-binding protein 7 | Q16270 | 0.621 | 0.008 |
| 38 | 3 | Megakaryocyte-associated tyrosine-protein kinase | P42679 | 0.817 | 0.008 |
| 28 | 4 | Plasminogen | P00747 | 0.609 | 0.008 |
| 36 | 5 | Agouti-related protein | O00253 | 0.479 | 0.008 |
| 18 | 3 | Hepatocyte growth factor | P14210 | 0.789 | 0.008 |
| 47 | 3 | A disintegrin and metalloproteinase with thrombospondin motifs 4 | O75173 | 0.774 | 0.007 |
| 48 | 2 | Serum paraoxonase/arylesterase 1 | P27169 | 0.979 | 0.006 |
| 51 | 3 | SLAM family member 5 | Q9UIB8 | 0.649 | 0.006 |
| 42 | 3 | Cytotoxic and regulatory T-cell molecule | O95727 | 0.613 | 0.006 |
| 54 | 2 | Protein Wnt-7a | O00755 | 0.914 | 0.006 |
| 37 | 2 | Vascular endothelial growth factor A isoform 121 | P15692 | 0.852 | 0.005 |
| 35 | 2 | Casein kinase II subunit alpha | P68400 | 0.838 | 0.005 |
| 53 | 2 | Fibroblast growth factor 2 | P09038 | 0.815 | 0.005 |
| 40 | 2 | Homeobox protein NANOG | Q9H9S0 | 0.744 | 0.005 |
| 55 | 2 | C-C motif chemokine 20 | P78556 | 0.62 | 0.004 |
| 49 | 1 | Kallikrein-5 | Q9Y337 | 1 | 0.003 |

^a^As defined in JMP: “The cluster’s proportion of variance explained by the first principal component among the variables in the cluster. If there is only one variable in the cluster, then this is 1. This statistic is based only on variables within the cluster rather than on all variables.”

^b^As defined in JMP: The overall proportion of variance explained by the cluster component. This is equivalent to using only the variables within each cluster to calculate the first principal component.

**Table S4. Clusters of serum T_2_-correlated SOMAscan biomarkers.**

| Cluster | Members | Most Representative Variable in Cluster | Uniprot ID | | Cluster Variation Explained^a^ | Total Variation Explained^b^ |
| --- | --- | --- | --- | --- | --- | --- |
| 1 | 15 | Semaphorin-6A | | Q9H2E6 | 0.69 | 0.038 |
| 2 | 15 | Limbic system-associated membrane protein | | Q13449 | 0.604 | 0.034 |
| 3 | 12 | Probetacellulin | | P35070 | 0.651 | 0.029 |
| 5 | 11 | Appetite-regulating hormone | | Q9UBU3 | 0.663 | 0.027 |
| 30 | 10 | Tumor necrosis factor ligand superfamily member 15 | | O95150 | 0.621 | 0.023 |
| 6 | 9 | Persephin | | O60542 | 0.635 | 0.021 |
| 9 | 10 | Latent-transforming growth factor beta binding protein 4 | | Q8N2S1 | 0.57 | 0.021 |
| 14 | 8 | Complement component C1q receptor | | Q9NPY3 | 0.628 | 0.019 |
| 4 | 9 | Angiotensinogen | | P01019 | 0.545 | 0.018 |
| 17 | 6 | CD70 antigen | | P32970 | 0.801 | 0.018 |
| 11 | 8 | Basal Cell Adhesion Molecule | | P50895 | 0.573 | 0.017 |
| 8 | 7 | C-C motif chemokine 5 | | P13501 | 0.654 | 0.017 |
| 18 | 6 | Calcineurin subunit B type 1 | | P63098 | 0.709 | 0.016 |
| 31 | 7 | Gamma-enolase | | P09104 | 0.607 | 0.016 |
| 27 | 7 | Antithrombin-III | | P01008 | 0.52 | 0.014 |
| 22 | 6 | Carbonic anhydrase 4 | | P22748 | 0.584 | 0.013 |
| 10 | 6 | Proteasome subunit alpha type-2 | | P25787 | 0.58 | 0.013 |
| 16 | 5 | Heme oxygenase 2 | | P30519 | 0.693 | 0.013 |
| 15 | 6 | Cytokine receptor-like factor 2 | | Q9HC73 | 0.566 | 0.013 |
| 45 | 5 | Ephrin type-A receptor 2 | | P29317 | 0.668 | 0.012 |
| 32 | 5 | Kallikrein-5 | | Q9Y337 | 0.667 | 0.012 |
| 23 | 5 | Angiopoietin-related protein 3 | | Q9Y5C1 | 0.66 | 0.012 |
| 20 | 6 | Metalloproteinase inhibitor 2 | | P16035 | 0.545 | 0.012 |
| 28 | 5 | Receptor-type tyrosine-protein kinase FLT3 | | P36888 | 0.653 | 0.012 |
| 12 | 5 | Semaphorin-3E | | O15041 | 0.645 | 0.012 |
| 40 | 6 | Muellerian-inhibiting factor | | P03971 | 0.53 | 0.012 |
| 13 | 5 | Proteasome activator complex subunit 3 | | P61289 | 0.635 | 0.012 |
| 21 | 5 | Quinone oxidoreductase-like protein 1 | | O95825 | 0.614 | 0.011 |
| 24 | 4 | Nidogen-2 | | Q14112 | 0.759 | 0.011 |
| 25 | 5 | Lactadherin | | Q08431 | 0.606 | 0.011 |
| 35 | 5 | Ubiquitin-conjugating enzyme E2 G2 | | P60604 | 0.581 | 0.011 |
| 38 | 4 | Serum paraoxonase/arylesterase 1 | | P27169 | 0.716 | 0.011 |
| 7 | 4 | Protein kinase C zeta type | | Q05513 | 0.683 | 0.01 |
| 37 | 5 | Complement C1s subcomponent | | P09871 | 0.516 | 0.01 |
| 36 | 3 | Glucokinase regulatory protein | | Q14397 | 0.82 | 0.009 |
| 44 | 3 | Nuclear receptor subfamily 1 group D member 1 | | P20393 | 0.743 | 0.008 |
| 19 | 3 | 40S ribosomal protein S3 | | P23396 | 0.725 | 0.008 |
| 42 | 3 | Endothelial cell-specific molecule 1 | | Q9NQ30 | 0.722 | 0.008 |
| 41 | 3 | Vascular cell adhesion protein 1 | | P19320 | 0.684 | 0.008 |
| 29 | 3 | Group IIE secretory phospholipase A2 | | Q9NZK7 | 0.676 | 0.008 |
| 39 | 3 | HERV-H LTR-associating protein 2 | | Q9UM44 | 0.648 | 0.007 |
| 26 | 3 | Trefoil factor 1 | | P04155 | 0.628 | 0.007 |
| 34 | 2 | Bactericidal permeability-increasing protein | | P17213 | 0.916 | 0.007 |
| 33 | 2 | Cytotoxic and regulatory T-cell molecule | | O95727 | 0.816 | 0.006 |
| 43 | 2 | Interferon alpha-7 | | P01567 | 0.73 | 0.005 |
| 46 | 1 | Fibrinogen | | P02671 P02675 P02679 | 1 | 0.004 |
| 47 | 1 | ATP synthase subunit O, mitochondrial | | P48047 | 1 | 0.004 |

^a^As defined in JMP: “The cluster’s proportion of variance explained by the first principal component among the variables in the cluster. If there is only one variable in the cluster, then this is 1. This statistic is based only on variables within the cluster rather than on all variables.”

^b^As defined in JMP: The overall proportion of variance explained by the cluster component. This is equivalent to using only the variables within each cluster to calculate the first principal component.
